# Influence of Cytochrome P450 2D6 Polymorphisms on the Efficacy of Oral Propranolol in Treating Infantile Hemangioma

**DOI:** 10.1155/2020/8732871

**Published:** 2020-03-07

**Authors:** Lidan Wang, Kai Zheng, Xinlin Li, Yang Wang, Qiong Xu

**Affiliations:** ^1^Department of Radiology, Wuhan Children's Hospital, Tongji Medical College, Huazhong University of Science & Technology, China; ^2^Department of General Surgery, Wuhan Children's Hospital, Tongji Medical College, Huazhong University of Science & Technology, China; ^3^Department of Clinical Pharmacology, Wuhan Children's Hospital, Tongji Medical College, Huazhong University of Science & Technology, China

## Abstract

**Objective:**

The aim of this study is to evaluate the association of genetic polymorphisms in Cytochrome P450 2D6(CYP2D6) and the change in VEGF levels with the response to propranolol in patients with Infantile hemangiomas (IH).

**Methods:**

IH patients who underwent over six months of propranolol therapy and received oral propranolol only were enrolled. The target dose of propranolol was 1 mg kg^−1^day^−1^. Deoxyribonucleic acid was obtained from venous blood leukocytes. Genotypes of CYP2D6 (rs1065852 and rs1135840) were tested by polymerase chain reaction (PCR) and by sequencing the products. Baseline serum VEGF and serum VEGF one month after treatment were measured. The clinical responses after six months of treatment were evaluated. Genotypes of CYP2D6 (rs1065852 and rs1135840) and VEGF levels were compared between good responders and poor-to-moderate responders.

**Results:**

72 patients were enrolled in the study. Patients with CYP2D6 (rs1135840) G/G homozygote had the highest response rate to propranolol. No significant association was found between the response rates and CYP2D6 (rs1065852) polymorphism. No significant differences were found in baseline serum VEGF, serum VEGF one month after treatment, and VEGF ratio between good responders and poor-to-moderate responders.

**Conclusion:**

The response to propranolol treatment in IH patients was associated with the gene polymorphism of CYP2D6 (rs1135840). A low-dose propranolol regimen was effective and safe in young infants with IH. The change of serum VEGF levels after one month's treatment could not be used to predict the response rate to propranolol.

## 1. Introduction

Infantile hemangiomas (IH) occur in 3% to 10% of infants [1]. Not all IH need treatment as IH have proliferative phase and spontaneous regression phase, and most IH can regress spontaneously. However, IH that can cause disfigurement and serious complications need treatment [2].

Oral propranolol is widely used in treating complicated IH [3]. However, the efficacy of propranolol on IH varies. Some patients failed propranolol treatment [4, 5]. Though the reported propranolol-resistant IH were rare, it is troublesome for patients who fail propranolol treatment as they often need to receive other individualized treatments such as surgical therapy [6], laser therapy [7], or injection of bleomycin [8].

During the growth phase, the growth speed of IH is different. Without effective intervention, the size of most IH (80%) will double, in 5% of patients, the size will triple, and in less than 5%, the size will dramatically extend [9]. When the effects of propranolol are evaluated after six months of therapy as in most studies, the lesions would have already become larger in the poor responders. If the alternative treatments are administered to the poor responders with larger lesions, it would be more painful for them because the wound of the alternative treatments would also be larger. Therefore, clinical or biological markers that can predict poor response to propranolol therapy will be useful for prognosis and enable early individualized treatments in the poor responders.

However, researches about markers of the poor response to propranolol are limited. A study tried to find the correlation between heart rate reduction after propranolol administration and the clinical outcome of propranolol therapy for IH. But no relationship was proved after six months of propranolol therapy [10].

It was reported that CYP2D6 was of great importance in propranolol metabolism [11]. Studies showed different CYP2D6 activities derived from the Cytochrome P450 2D6 (CYP2D6) polymorphism [12, 13]. Poor metabolizers of the CYP2D6 C/T188 genotypes had less activity, and therefore, the plasma concentrations of propranolol were higher in patients with poor metabolizers than in those with extensive metabolizers [14]. It is possible that the CYP2D6 genetic polymorphisms may affect the outcome of propranolol therapy for IH. However, studies about the association between CYP2D6 genetic polymorphisms and the clinical effects of propranolol on IH are rare.

Increased vascular endothelial growth factor (VEGF) level is considered as one of the mechanisms of IH. Serum VEGF levels increased in the growth phase of IH and significantly decreased in the involuting phase [15]. It was shown that propranolol inhibited VEGF expression [16]. A study showed that the decreases of serum VEGF concentrations were more obvious after one month of propranolol therapy than those after three months of therapy [17]. Another study showed that the biggest change measured in Hemangioma Activity Score (HAS) occurred after one month of propranolol treatment [18]. Maybe the changes in VEGF after one month of treatment would be correlated with the response rate to propranolol and could be used to predict the response to propranolol.

In this study, we conducted a prospective study in children with IH to investigate the relationship between CYP2D6 (rs1065852 and rs1135840) gene polymorphisms and the efficacy of propranolol on children with IH. The predictive value of the change in serum VEGF was also investigated.

## 2. Materials and Methods

### 2.1. Patients

Between June 2017 and January 2019, children with newly diagnosed IH who underwent over six months of propranolol therapy and received oral propranolol only were enrolled in the study. The study was approved by the Research Ethics Board of Wu Han Children's Hospital and was conducted in conformity with the ethical principles of the Declaration of Helsinki. Consent of the patients' legal guardians was obtained.

### 2.2. Treatment

Before oral propranolol treatment, echocardiography and electrocardiography were performed. Patients with mixed or deep IH underwent magnetic resonance imaging (MRI). Parents or guardians were informed of signs of adverse drug reactions (including hypoglycemia, hypotension, bradycardia, and bronchospasm) and precautionary measures.

Propranolol tablet (10 mg a tablet) was administered once a day orally to patients in the morning immediately before feeding. The initial dose was 0.5 mg kg^−1^day^−1^, and the dose increased to the target dose (1 mg kg^−1^day^−1^) on the eighth day.

Monthly visits were conducted after reaching the target dose. The dose of propranolol was altered based on weight change at each visit.

Clinical data were recorded at each visit by a clinician including age, gender, body weight, height, serum creatinine (Scr), alanine aminotransferase (ALT), blood glucose, heart rate, IH classification, number of lesions, location, and propranolol dosage. The size of the hemangioma lesion was measured and documented at each visit. Digital photographs were taken. Changes in color and lesion size and hemangioma development (improvement, stabilization, or aggravation) were assessed and compared with previous visits. Patients with mixed or deep IH underwent MRI after six months of treatment. The clinical responses after six months of treatment were evaluated. Treatments with complete or approximately complete resolutions of the lesions were considered successful ones. The approximately complete resolution was defined as a slight degree of IH lesion [19]. Patients with a successful treatment were categorized as good responders. The rest of the patients were categorized as poor-to-moderate responders.

As superficial IH occur earlier and involution of them begin sooner than deeper ones [7], the differences of the clinical IH types (superficial, deep, and mixed components) between good responders and poor-to-moderate responders were compared. As segmental hemangiomas needed longer therapy and had a poorer overall outcome [20], to exclude the influence of the longer treatment durations of segmental hemangiomas on the outcome of our study, the constituent ratios of focal, segmental, and multifocal hemangiomas were also compared between good responders and poor-to-moderate responders.

After six months of treatment, propranolol was gradually withdrawn in patients with complete resolution. Treatment continued to 12 months if the regression of the lesion was lower than 90%. No patients received propranolol therapy for over 12 months.

Blood samples were drawn before and at one month after the initiation of propranolol treatment. Serum samples were obtained and stored at −20°C.

### 2.3. Determination of Serum VEGF

Serum VEGF levels were determined by Human VEGF Enzyme-Linked Immunosorbent Assay (ELISA) Kits EK183-96 (Multisciences (Lianke) Biotech, Co., Ltd., Hangzhou, China). The detection range of the ELISA Kit was 1-1000 pg ml^−1^. All serum VEGF levels in the study were within the detection range of the Kits and were tested in accordance with the instruction of the Kit.

Baseline serum VEGF levels were the VEGF levels measured one day before the propranolol administration. Serum VEGF ratios were got by dividing the serum VEGF levels one month after the treatment by the baseline VEGF levels.

### 2.4. CYP2D6 SNP Genotyping

Deoxyribonucleic acid was obtained from venous blood leukocytes. Genotypes of CYP2D6 (rs1065852 and rs1135840) were tested by polymerase chain reaction (PCR) and by sequencing the products. The PCR primers were designed and synthesized by Shanghai Thermo Fisher Scientific (China) Co., Ltd. PCR was performed by Bio-rad S1000 Thermal Cycler. Genotyping was performed by sanger sequencing.

### 2.5. Statistical Analysis

Statistical analysis was performed using the Statistical Package for the Social Sciences (SPSS version 21.0, SPSS Inc., Chicago, IL, USA). The normality of the distributions of continuous variables was checked by Kolmogorov-Smirnov test. Normally distributed continuous variables were compared using a Student's *t* test. Nonnormally distributed continuous variables data were compared with a Mann-Whitney *U* test. Categorical data were compared using a chi-square test. Variables with a two-tailed *p* value <0.05 were considered statistically significant.

## 3. Results

72 patients were enrolled. The patients' demographic characteristics and serum VEGF levels are presented in Table 1. 56 patients were female (77.8%), and 16 were male (22.2%). The median age of the patients at propranolol initiation was 65.5 days (range, 33-274 days). 52 patients were younger than 3 months old at propranolol initiation.

**Figure 1:**
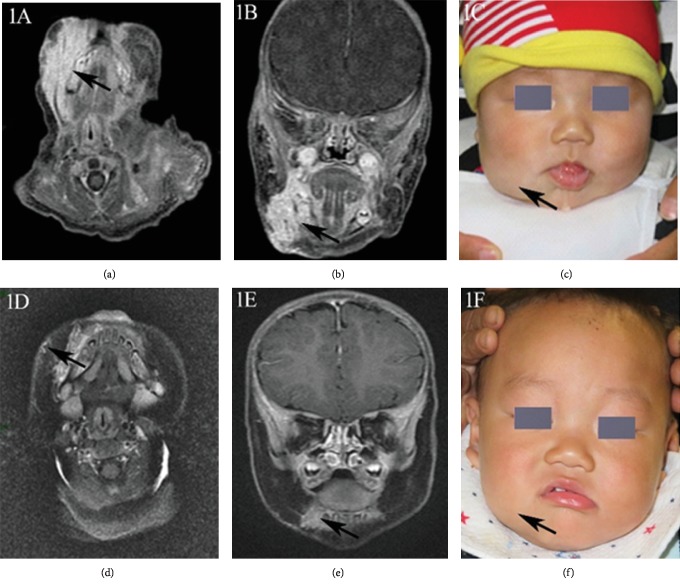
An 11-week-old boy with a deep IH on the right maxillofacial region. (a–c) Before propranolol treatment, (a–b) axial and coronal MRI T_1_WI enhancement showed obvious high signal of the right maxillofacial hemangioma. (d–f) After 8 months of propranolol treatment, (d, e) axial and coronal MRI T_1_WI enhancement showed only a few residual lesions.

**Figure 2:**
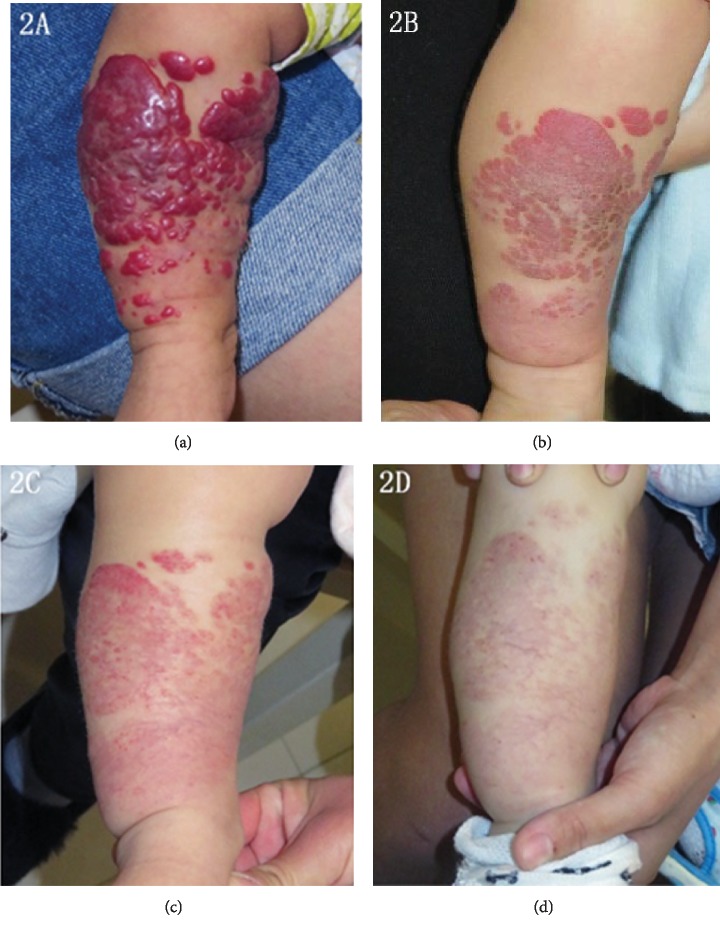
A 9-week-old girl with an IH on the right leg. (a) 1 day before propranolol treatment, (b) after 3 months of propranolol treatment, (c) after 6 months of propranolol treatment, (d) 12 months after propranolol treatment.

**Figure 3:**
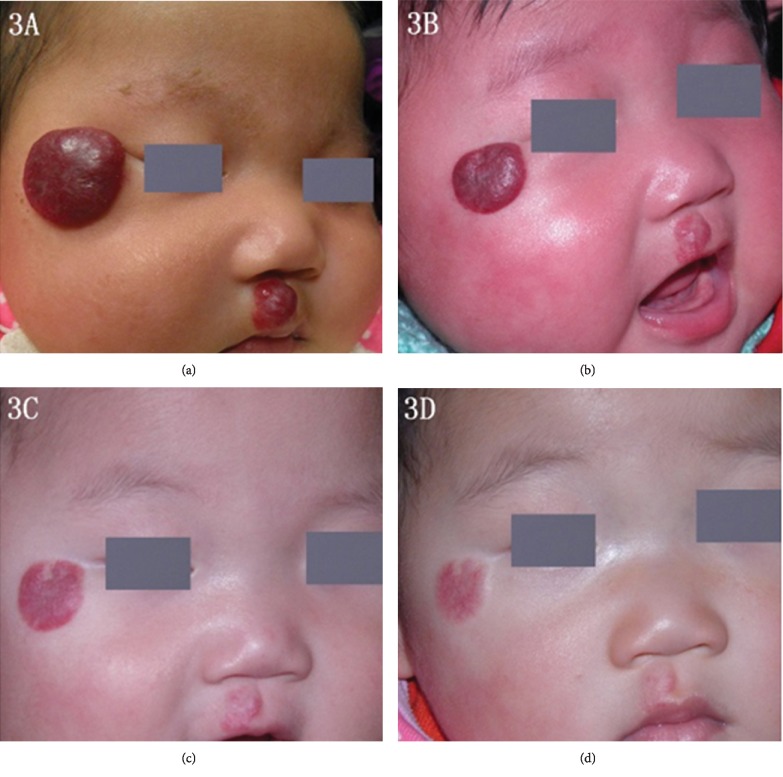
A 10-week-old girl with an IH on the right cheek. (a) 1 day before propranolol treatment, (b) after 3 months of propranolol treatment, (c) after 6 months of propranolol treatment, (d) 12 months after propranolol treatment.

IH can be morphologically categorized into 4 groups: localized, segmental, indeterminate, and multifocal hemangiomas [9]. However, in our study, no patients presented indeterminate hemangiomas. 51 patients (70.8%) had localized lesions, 13 patients (18.1%) had segmental lesions, while 8 patients (11.1%)had multifocal lesions. The lesions were located on facial areas in 46 patients (63.9%) and on nonfacial areas in 26 (36.1%). 54 patients had good responses to propranolol after six months of therapy. Typical cases are shown in Figures 1–3. There were no significant differences between good responders and poor-to-moderate responders in gestationally corrected age at inclusion, gender, body weight, height, ALT, Scr, baseline heart rate, baseline blood pressure, morphologic classification, and the clinical IH types (superficial, deep, and mixed components).

Baseline serum VEGF, serum VEGF one month after treatment, and VEGF ratio were compared between good responders and poor-to-moderate responders, but no significant differences were found.

### 3.1. Patients' Genotype Distribution

The allelic frequencies of CYP2D6 (rs1065852 and rs1135840) were consistent with the Hardy-Weinberg equilibrium.

The genotype of rs1065852 and rs1135840 and their associations with the treatment responses were shown in Table 2, respectively.

After six months of treatment, no significant association was found between the response rates and CYP2D6 (rs1065852) polymorphism. Patients with the G/G genotype of CYP2D6 (rs1135840) had the highest response rate, while those with the C/C type had the lowest response rate.

### 3.2. Side Effects

No severe side effects including pulmonary symptoms, hypoglycemia, or bradycardia were found by physicians, parents, or guardians during the treatment. No patients needed any dose reduction of propranolol, and no one withdrew from treatment because of adverse reactions. Five children experienced slight diarrhea at the initiation of the administration of propranolol, and diarrhea disappeared in three days in most cases.

## 4. Discussion

Oral propranolol is now widely used in treating complicated IH. However, some patients do not show a good response to propranolol and need additional treatment other than propranolol alone. Nowadays, there is no satisfying marker that can predict poor response to propranolol treatment.

In the present study, the impact of CYP2D6 (rs1065852 and rs1135840) gene polymorphisms on the efficacy of oral propranolol in treating IH was investigated. It showed that the patients with rs1135840 (CYP2D6) G/G homozygotes had the highest response rate, and the patients with the C/C type had the lowest response rate after six months of treatment. The response to propranolol treatment in IH patients was related to gene polymorphism of rs1135840 (CYP2D6). A study investigating the relationship between polymorphisms of Cytochrome P450 2D6 and blood hydroxychloroquine levels in patients with systemic lupus erythematosus showed that patients with rs1135840 G/G homozygotes had decreased CYP2D6 activity and lower concentrations of metabolites [21]. The highest response rate in patients with rs1135840 (CYP2D6) G/G homozygote in this study might be associated with a higher serum propranolol concentration. We speculate that for patients with rs1135840 G/C or C/C genotype, the dose of propranolol could be individualized provided the monitoring of serum propranolol concentrations. However, the speculation needs to be further investigated.

The reported propranolol target doses were diverse, and the optimal doses were inconsistent in different reports. A study showed that at the dose of 3 mg kg^−1^day^−1^, patients could get the optimal response [19]. Another study showed that no significant difference was found in the treatment response in different doses, while the side-effects of propranolol were found to be associated with a higher dose of propranolol [22]. It seems that lower propranolol dose is safer for infants with IHs. There have been studies showing the effectiveness of low-dose propranolol for IHs [23, 24], and the dose of 1.0 mg kg^−1^day^−1^ was proved to be successful in treating IHs^24^. In our study, 1 daily administration of propranolol was chosen for three main reasons. Firstly, a study showed that the administration of propranolol once a day at a dose of 1.0 mg kg^−1^day^−1^ was effective in the treatment of superficial IH [25]. Secondly, in our previous clinical practice, we found that 1 daily administration of propranolol at the dose of 1.0 mg kg^−1^day^−1^was also effective in treating other types of IHs including deep IHs and mixed IHs. Thirdly, there is no propranolol oral solution available in China. As the target dose of propranolol in our study was relatively low, it would be difficult for parents to divide the dose accurately to twice or three times a day. 1 daily administration could increase patient compliances. The response rate in our study after six months of treatment was satisfying. The side effects in this study were slight.

A study conducted in infants aged less than four months showed that propranolol could stop the growth of IH, while a mild growth of IH was observed in the placebo group [26]. A study about the early growth characteristics of IHs showed that most rapid growth of IH appeared between 5.5 and 7.5 weeks of age [27]. For patients who need to be treated, oral propranolol is proposed to be administered as early as possible to prevent the occurrence of potential complications [28]. In our study, 52 patients were younger than 3 months old at the propranolol initiation. Most patients received the administration of propranolol about one month after the occurrence of IH. No severe side effects were noticed by physicians, parents, or guardians during the treatment. The low-dose propranolol regimen was effective and safe in young infants with IH.

Another finding in the present study is that the changes in serum VEGF levels after one month of therapy could not be used to predict the response rate to propranolol. The exact mechanisms of propranolol in treating IH are still not clarified, but propranolol was found to be able to regulate hemangioma cell proliferation through the impact on the VEGF pathway [29]. In the present study, VEGF levels one month after the initiation of treatment decreased compared with their baseline VEGF which was consistent with a previous study [17]. However, no difference in percentage VEGF changes after one month of treatment between the poor-to-moderate responders and the good responders were found.

There were a few limitations in the present study. Firstly, the study included a small number of patients. The findings need to be proved in studies with a larger number of patients. Secondly, there was no placebo group in this study. Though the study was a prospective one, no patients' guardians were willing to take a placebo during the treatment.

## 5. Conclusion

The response to propranolol treatment in IH patients was associated with the gene polymorphism of CYP2D6 (rs1135840). A low-dose propranolol regimen was effective and safe in young infants with IH. The change of serum VEGF levels after one month's treatment could not be used to predict the response rate to propranolol.

## Figures and Tables

**Table 1:** Patients' characteristics and serum VEGF levels.

Characteristics	Total patients	Good responders	Poor-to-moderate responders	*p* ^#^
Patient number *n* (%)	72	54 (75)	18 (25)	
Corrected age at inclusion (days) [median (IQR)]	65.5 (48.2-93.8)	70.0 (47.5-100.0)	53.5 (46.0-88.0)	0.191^b^
Gender (male/female) (*n*)	16/56	12/42	4/14	1.0^c^
Body weight (kg) [median (IQR)]	5.5 (4.8-6.4)	5.5 (5.0-6.5)	5.5 (4.5-6.1)	0.399^b^
Height (cm) [mean (SD)]/[median (IQR)]	57.32 (4.71)	57.0 (54.0-61.1)	56.0 (53.8-58.3)	0.344^b^
Blood glucose (mmol L^−1^) [mean (SD)]	4.93 (0.36)	4.95 (0.37)	4.86 (0.32)	0.366^a^
ALT (U L^−1^) [mean (SD)]	30.00 (7.95)	30.28 (7.73)	29.17 (8.78)	0.611^a^
Scr (*μ*mol L^−1^) [mean (SD)]	21.00 (3.50)	20.84 (3.42)	21.50 (3.80)	0.491^a^
Baseline heart rate (beats per minute)[mean (SD)]	129.79 (6.53)	129.46 (6.35)	130.78 (7.14)	0.463^a^
Clinical IH types				0.905^c^
Superficial component *n* (%)	39 (54.2)	30 (55.6)	9 (50.0)	
Deep component *n* (%)	4 (5.6)	3 (5.6)	1 (5.6)	
Mixed component *n* (%)	29 (40.3)	21 (38.9)	8 (44.4)	
Morphologic classification				0.517^c^
Localized *n* (%)	51 (70.8)	36 (66.7)	15 (83.3)	
Segmental *n* (%)	13 (18.1)	11 (20.4)	2 (11.1)	
Multifocal *n* (%)	8 (11.1)	7 (13.0)	1 (5.6)	
Baseline serum VEGF (pg ml^−1^) [median (IQR)]	202.69 (149.72-251.54)	201.35 (156.16-240.73)	213.22 (114.75-255.08)	0.682^b^
Serum VEGF 1 month after treatment (pg ml^−1^) [median (IQR)]	168.07 (101.91-222.31)	169.35 (105.37-219.71)	154.59 (88.30-253.27)	0.886^b^
Serum VEGF ratio [median (IQR)]	0.82 (0.65-1.04)	0.81 (0.62-1.04)	0.84 (0.71-1.04)	0.603^b^

^#^Comparisons between good responders and poor-to-moderate responders. ^a^Normally distributed continuous variables were reported as means with standard deviations (SD) and compared with Student's *t* test. ^b^Nonnormally distributed continuous variables data were reported as medians with interquartile range (IQR) and compared with Mann-Whitney *U* test. ^c^Categorical data were reported as proportions(*n* with %)and compared using chi-square test.

**Table 2:** Associations between CYP2D6 genotypes and the treatment responses.

	*N*	Good responders	Poor-to-moderate responders	*p*
rs1065852				0.541
G/G *n* (%)	17	11 (64.7)	6 (35.3)	
A/G *n* (%)	39	30 (76.9)	9 (23.1)	
A/A *n* (%)	16	13 (81.3)	3 (18.8)	
rs1135840				0.031
C/C *n* (%)	11	6 (54.5)	5 (45.5)	
C/G *n* (%)	31	21 (67.7)	10 (32.3)	
G/G *n* (%)	30	27 (90.0)	3 (10.0)	

Categorical data were reported as proportions (*n* with %)and compared using chi-square test.

## Data Availability

The data used to support the findings of this study are available from the corresponding author upon request.
